# Supplementary material for: Vertical redistribution of salt and layered changes in global ocean salinity
Source: Nat Commun. 2019 Aug 1;10:3445. doi: 10.1038/s41467-019-11436-x (PMC6671951; doi:10.1038/s41467-019-11436-x)
Supplement: Supplementary file 1 — SUPPLEMENTARY INFO [file 41467_2019_11436_MOESM1_ESM.pdf]

Supplementary Information for

Vertical Redistribution of Salt and Layered Changes in Global Ocean Salinity

Chao Liu[1], Xinfeng Liang[1*], Rui M. Ponte[2], Nadya Vinogradova[3,4], Ou Wang[5]

Supplementary Figures

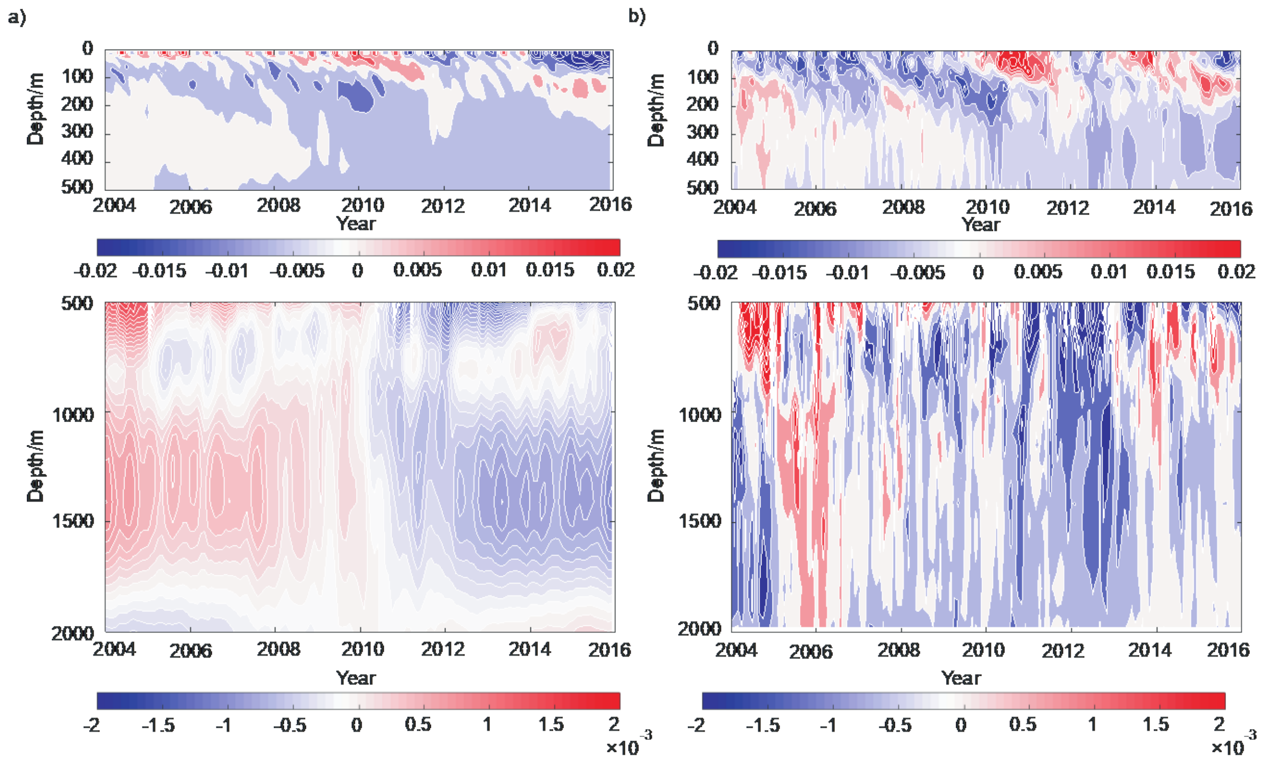

Supplementary Figure 1: Temporal evolution of the ocean salinity from ECCO and Argo. Hovmoller diagram of the horizontally averaged ocean salinity anomaly (unit: psu) from **(a)** ECCO v4r3 and **(b)** Argo over the overlapping period (2005-2015).

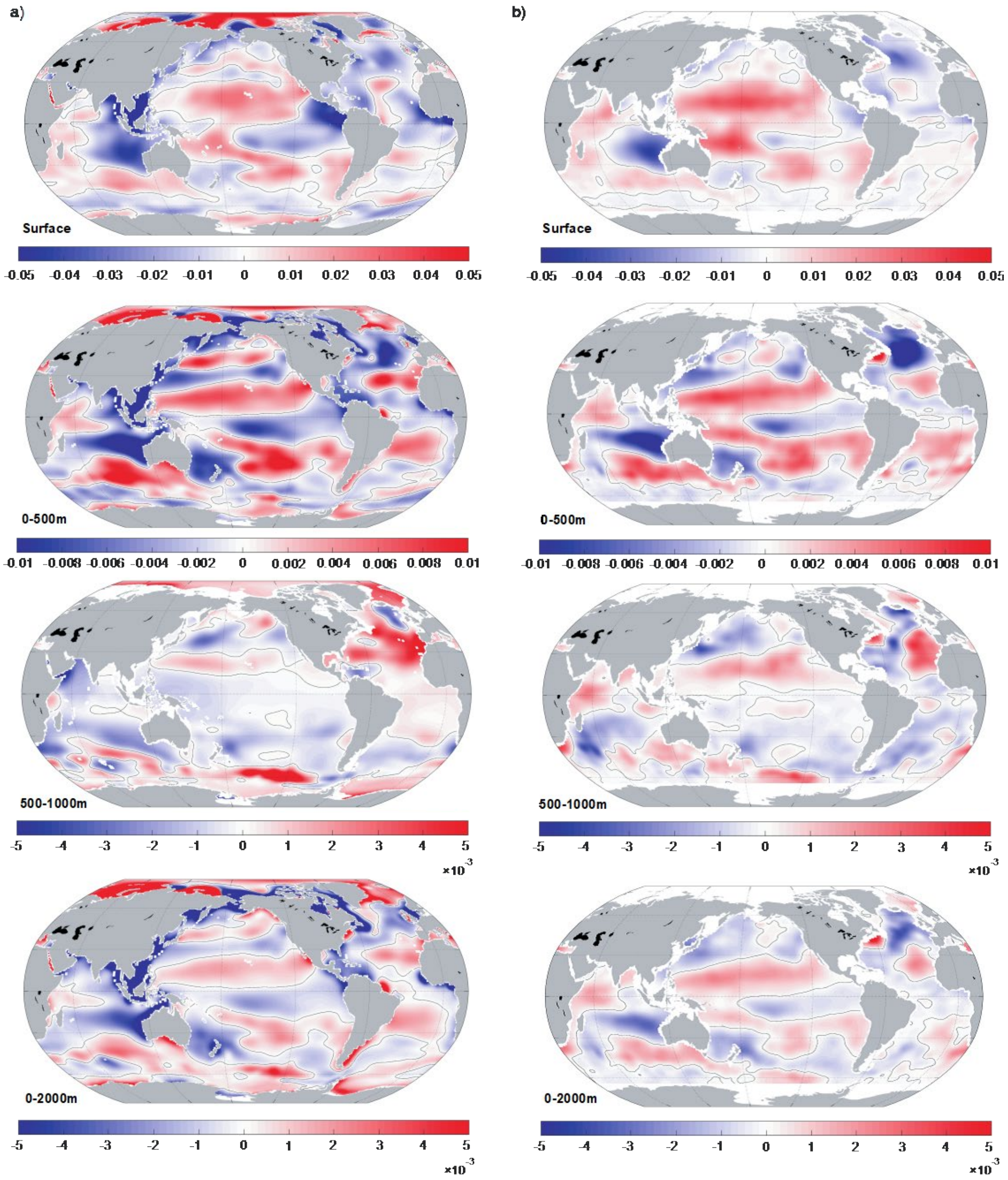

Supplementary Figure 2: Decadal trends of ocean salinity from ECCO and Argo. Trends of ocean salinity (psu year^{-1}) over the overlapping period (2005-2015) in sampled layers from ECCO v4r3 (**a, left**) and Argo (**b, right**). Values of zero are marked as black contours. Note the varying scale in different panels.

Supplementary Note 1

Previous studies have shown that the ECCO v4 estimates agree well with various observations, such as sea level anomalies[1], sea surface temperature[2], subsurface hydrography[3], and the Atlantic Meridional Overturning Circulation (AMOC[4]). Other quantities (e.g., vertical velocity), for which no corresponding global observations are available, have been analyzed and found to be at least physically plausible[5,6]. Here we briefly assess the quality of the salinity estimates from ECCO v4 by comparing them with existing *in situ* salinity observations and previous studies.

We first compare the ECCO v4r3 estimate with the global gridded Argo dataset from Scripps Institution of Oceanography (the Roemmich-Gilson Argo Climatology) within the overlapping period (2005-2015). The upper ocean salinity changes from ECCO v4r3 and Argo show a number of consistent temporal and spatial patterns (Supplementary Figure 1). For instance, the upper ocean salinity from both Argo and ECCO shows strong interannual variability that is related to the significant ENSO events. Although the agreement between ECCO and Argo below 500 m is not as good as the upper 500 m, both estimates display a freshening as well as a few extreme events (e.g., salinification at 700 m around the year 2014). The spatial patterns of the long-term salinity changes from ECCO and Argo also show satisfactory agreement in all the examined layers (Supplementary Figure 2).

The ECCO estimate also displays consistent patterns with previous studies that are based on observations or reanalysis products but on different periods of analysis[7-9]. For example, for the Atlantic Ocean, the ECCO estimate shows strong salinification in the surface and upper ocean at high latitudes in both the North and South Atlantic Ocean, as well as freshening at the midlatitudes. The regions of salinification in the Atlantic are generally consistent with the long-term salinity changes shown in Argo and EN3 data[8,9]. For the Indo-Pacific, significant

salinification appears in the western tropical Pacific Ocean and freshening occurs in the southeastern tropical Indian Ocean (Supplementary Figure 2). Such features are consistent with results obtained from the Argo data[10]. Furthermore, for the intermediate water, the freshening (between 500-2000 m) in the South Atlantic Ocean is also captured by Argo after 2000[11].

For the deep and abyssal oceans, the existing studies are much limited to particular regions, but the consistency remains. For instance, the ECCO estimate shows clear freshening below 2000 m in the North Atlantic Ocean (Figure 3f), which is consistent with previous studies based on the WOCE measurements[12]. Also, a freshening can be found in the abyssal southeastern Indian Ocean (below 2000 m, Figure 3f), which is in accord with a previous study based on WOCE[13]. We also found a salinity increase in the Southern Ocean adjacent to the Antarctic Continent at almost all the examined layers except the abyssal ocean (>4500 m, not shown). A similar pattern was discovered by previous studies[14], and the wind-driven northward transport of sea ice could contribute to such changes.

Supplementary References

1. Forget, G., and Ponte, R. The partition of regional sea level variability. *Prog Oceanogr.* **137.** 173-195 (2015).
2. Buckley, M., Ponte, R., Forget, G., and Heimbach, P. Low-Frequency SST and Upper-Ocean Heat Content Variability in the North Atlantic. *J. Clim.* **27.** 4996-5018 (2014).
3. Forget, G., Campin, J., Heimbach, P., Hill, C., Ponte, R., and Wunsch, C. ECCO version 4: an integrated framework for non-linear inverse modeling and global ocean state estimation. *Geosci. Model Dev.* **8.** 3071-3104 (2015).
4. Wunsch, C., and Heimbach, P. Two Decades of the Atlantic Meridional Overturning Circulation: Anatomy, Variations, Extremes, Prediction, and Overcoming Its Limitations. *J. Clim.* **26.** 7167-7186 (2013).
5. Forget, G., Ferreira, D., and Liang, X. On the observability of turbulent transport rates by Argo: supporting evidence from an inversion experiment. *Ocean Sci.* **11.** 839-853 (2015).
6. Liang, X., Spall, M., and Wunsch, C. Global Ocean Vertical Velocity from a Dynamically Consistent Ocean State Estimate. *J. Geophys. Res -Oceans.* **122.** 8208-8224 (2017).
7. Boyer, T., Levitus, S., Antonov, J., Locarnini, R., and Garcia H. Linear trends in salinity for the World Ocean, 1955–1998. *Geophys. Res. Lett.* **32.** (2005).
8. Durack, P., and Wijffels, S. Fifty-Year Trends in Global Ocean Salinities and Their Relationship to Broad-Scale Warming. *J. Clim.* **23.** 4342-4362 (2010).
9. Skliris, N., Marsh, R., Josey, S., Good, S., Liu, C., and Allan, R. Salinity changes in the World Ocean since 1950 in relation to changing surface freshwater fluxes, *Clim. Dyn.* **43.** 709-736 (2014).
10. Du, Y., Zhang, Y., Feng, M., Wang, T., Zhang, N., and Wijffels, S. Decadal trends of the

upper ocean salinity in the tropical Indo-Pacific since mid-1990s. *Sci Rep.* **5.** 16050 (2015).

11. Yao, W., Shi, J., and Zhao, X. Freshening of Antarctic Intermediate Water in the South Atlantic Ocean in 2005–2014. *Ocean Sci.* **13.** 521-530 (2017).
12. Dickson, B., Yashayaev, I., Meincke, J., Turrell, B., Dye, S., and Holfort, J. Rapid freshening of the deep North Atlantic Ocean over the past four decades. *Nature.* **416.** 832-837 (2002).
13. Johnson, G., Purkey, S., and Bullister, J. Warming and Freshening in the Abyssal Southeastern Indian Ocean. *J. Clim.* **21.** 5351-5363 (2008).
14. Haumann, F., Gruber, N., Munnich, M., Frenger, I., and Kern, S. Sea-ice transport driving Southern Ocean salinity and its recent trends. *Nature.* **537.** 89-92 (2016).
